# Supplementary material for: Smooth Interpolating Curves with Local Control and Monotone Alternating Curvature
Source: Comput Graph Forum. 2022 Oct 6;41(5):25–38. doi: 10.1111/cgf.14600 (PMC9827861; doi:10.1111/cgf.14600)
Supplement: Supplementary file 1 — Supplement Material [file CGF-41-25-s001.zip › Local-Smooth-Interpolating-MonoCurvature/extern/clothoids/submodules/Utils/docs/api-cpp/file_view_hierarchy.html]

File Hierarchy — Utils v1.2

### Navigation

- index
- toc
- Utils »
- File Hierarchy

# File Hierarchy¶

- - Directory Utils
    - File Console.hxx
    - File Malloc.hxx
    - File Numbers.hxx
    - File Quaternion.hxx
    - File rang.hxx
    - File Table.hxx
    - File ThreadPool.hxx
    - File TicToc.hxx
    - File Token.hxx
    - File Trace.hxx
    - File Utils.hxx
  - File Console.cc
  - File CPUinfo.cc
  - File format.cc
  - File Malloc.cc
  - File Numbers.cc
  - File os.cc
  - File rang.cc
  - File Table.cc
  - File TicToc.cc
  - File Token.cc
  - File Trace.cc
  - File Utils.cc
  - File Utils.hh
  - File Utils\_CPU\_info.hh
  - File Utils\_zstr.hh

### Quick search

### Table of Contents

- rang - Colors for your Terminal
- Zstr
- C++ API

«
hide menu

menu
sidebar
»

### Navigation

- index
- toc
- Utils »
- File Hierarchy

© Copyright 2021, Enrico Bertolazzi.
Created using Sphinx 4.2.0.
